# Mr. Dobson on the Structure and Functions of the Spleen

**Published:** 1830-10-01

**Authors:** 


### XXXIX.

### Mr. Dobson on the Structure and Function of the Spleen.

We had hardly printed our remarks on Sir Anthony Carlisle's speculations on the spleen and thyroid gland, when another pamphlet on the same subject was laid on our table. Mr. Dobson went to work in his investigation by a series of experiments on animals—a mode of procedure which is far from unobjectionable; but which, at all events, is superior to the closet speculations of Sir Anthony. If they are tinctured with *cruelty* towards dogs, they are free from *spleen* towards man.

Mr. D's first object of inquiry was to ascertain the changes produced on the spleen by the digestive process, and the exact period, after ingestion, at which such changes take place. We need not detail the experiments, but only their results. From these it appears that, till the expiration of three hours after a full meal, the dog's spleen does not present any visible alteration. At the end of four hours it is much enlarged—and in five hours, it attains its maximum, after which it gradually decreases in bulk for 12 hours.

In the next train of experiments, Mr. Dobson removed the spleens of dogs.

"Exp. I. The spleen of a dog was removed; the animal apparently suffered little from the operation. On the following day I gave it a quantity of food; it ate voraciously; for three hours after no perceptible alteration was produced; but in *four hours after,* indications of uneasiness were shown; the animal became restless, and lastly sunk into a nearly torpid state; it was often moaning, the pupils were dilated,—the heart labouring; there was frequent micturition; the respiration was exceedingly laborious, and, in short, there was every mark of plethora, or over-fulness of the vascular system. In the course of two hours from this period, the animal began to recover; and in about three hours these symptoms had subsided, considerable languor remained. The animal took a large meal twice or thrice in twenty-four hours, and after each, precisely similar effects were presented. The animal became more feeble daily,—in a month after the operation, it died.

Exp. II. I next removed the spleen from another dog, but instead of giving full meals, as in the last experiment, I gave a small quantity of food every hour, or every two hours. The animal ate voraciously; no unpleasant symptoms occurred,—this plan was pursued for three weeks, when the animal to all appearance was quite well; in fact, it became fat; the ligature upon the splenic artery had come away, and the wound in the abdomen healed. I then commenced giving full meals twice in twenty fours, the same train of symptoms followed each meal, and at the same period, as in the last experiment, though perhaps not so urgent: the animal died in a month from the commencement of this plan of feeding.

In both dogs I observed that the intestinal evacuations were of a *lighter* colour than natural. On examining the body of each after death, a small quantity of limpid serum was contained in the bag of the Tunica Arachnoides, and more than a natural quantity in the lateral ventricles; the veins of the brain were in a highly congested state; the abdominal viscera presented no unnatural appearances, but the portal system of veins was much gorged with blood."

It may be stated that, generally speaking, the dog finishes its first digestion in three or three hours and a half—and, consequently, the turgidity of the spleen appears to commence when the stomach is emptying itself into the duodenum. The following is the deduction which Mr. D. draws from his first series of experiments, viz.

"*That the spleen acts as a reservoir for containing the additional quantity of blood which the vascular system has received, by means of the nutritive pro-*

---

markably correct. The case is, on the whole, a rare and curious one. The patient was under the care of the editor, but other avocations prevented his attending with regularity and minuteness to the symptoms, which will account for the concise and abbreviated form of the notes.

*cess.* It is evident from the remarks on chylification there is a greater quantity of blood in the system at *five hours* after a meal than at any other period; and as we have premised, that the blood-vessels are not capable of containing this increase with impunity, I infer, that *the spleen serves as a reservoir to hold this surplus*, because, at the time the chylifactive process is at an end, the spleen is found distended with blood. Then, as detailed in the third experiment, at twelve hours after a meal, the spleen was small, and contained very little blood: the reason of this phenomenon is obvious; at five hours after a meal, the nutritive process is nearly completed; at five hours after a meal, the spleen arrives at its maximum size: now, as secretion goes on in the various emunctories, there must consequently be a reduction of the circulating mass; and to compensate for this, blood is simultaneously expelled from the spleen, so that in twelve hours the whole is removed; no more circulating through that organ than is necessary for its support."

It was seen that when the spleen was removed, and the dog kept on full meals of animal food, he died: but when kept on abstemious fare, he went on very well. Mr. D. infers from this, that—"the *circulatory vessels are capable of containing only a certain quantity of blood with impunity, and that when an increase in the volume is produced, as after digestion, the spleen performs the office of a reservoir to receive the surplus;* they show also, that when the fluid contained in the vessels becomes reduced in quantity, as from bleeding, the spleen affords a supply, so as to enable the various organs to perform their necessary offices: and further, they afford collateral evidence of the spleen being more elastic than the blood-vessels." Mr. D. conceives that when we drink a bottle or two of wine, or large quantities of malt-liquor, the spleen performs the office of reservoir for the time. He had twice an opportunity of examining the spleens of great ale-bibbers, and found them very much enlarged—in one case, so enormously so, as to give the idea of "a bladder half filled with oil."

The pathology of the spleen has afforded no direct indication of its function. When the liver is diseased, the function of that viscus generally shews the existence of the malady—but, in reference to the spleen, we have no such tangible data afforded as a clue. The enlargement of the spleen, after protracted agues and remittent fevers, may be explained by the congestion of blood in this, and indeed in some other organs, during the cold stages of these diseases. The practical inferences drawn by our author are the following.

"I. That the quantity of fluid usually taken into the system at one time, is greater than the apparatus is capable of containing with impunity; and, in consequence of this, excited vascular action, with all its train of morbid consequences, is a common effect.

II. That in disorders affecting the spleen, as in Intermittent Fever, and as well of the whole vascular system, the practice of giving *large quantities of fluid*, is not only unphilosophical, but decidedly injurious."

We have thus laid before our readers the substance of Mr. Dobson's pamphlet, and shall only observe that the theory proposed, and the data on which this theory is based, are much more entitled to respect than those of Sir Anthony Carlisle, notwithstanding the "ENLARGED INTELLECT" which he modestly claims for himself and a few of his own rank.

